# Mutations in PI3K/AKT pathway genes and amplifications of *PIK3CA* are associated with patterns of recurrence in gastric cancers

**DOI:** 10.18632/oncotarget.6641

**Published:** 2015-12-17

**Authors:** Wen-Liang Fang, Kuo-Hung Huang, Yuan-Tzu Lan, Chien-Hsing Lin, Shih-Ching Chang, Ming-Huang Chen, Yee Chao, Wen-Chang Lin, Su-Shun Lo, Anna Fen-Yau Li, Chew-Wun Wu, Shih-Hwa Chiou, Yi-Ming Shyr

**Affiliations:** ^1^ Division of General Surgery, Department of Surgery, Taipei Veterans General Hospital, Taipei City, Taiwan; ^2^ School of Medicine, National Yang-Ming University, Taipei City, Taiwan; ^3^ Institute of Clinical Medicine, School of Medicine, National Yang-Ming University, Taipei City, Taiwan; ^4^ Division of Colon & Rectal Surgery, Department of Surgery, Taipei Veterans General Hospital, Taipei City, Taiwan; ^5^ Genome Research Center, National Yang-Ming University, Taipei City, Taiwan; ^6^ Division of Hematology and Oncology, Department of Medicine, Taipei Veterans General Hospital, Taipei, Taiwan; ^7^ Institute of Biomedical Sciences, Academia Sinica, Taipei, Taiwan; ^8^ Institute of Biotechnology in Medicine, National Yang-Ming University, Taipei, Taiwan; ^9^ National Yang-Ming University Hospital, Yilan City, Taiwan; ^10^ Department of Pathology, Taipei Veterans General Hospital, Taipei City, Taiwan; ^11^ Department of Medical Research and Education, Taipei Veterans General Hospital, Taipei City, Taiwan; ^12^ Institute of Pharmacology, National Yang-Ming University, Taipei City, Taiwan

**Keywords:** PI3K/AKT pathway, PIK3CA amplifications, recurrence pattern, diffuse-type, prognosis

## Abstract

Mutations in genes involved in the PI3K/AKT pathway and amplifications of the *PIK3CA* gene in gastric cancer and their associations with clinicopathological characteristics and EBV infection were analyzed in this study. A total of 431 patients with gastric adenocarcinomas were enrolled, and 39 mutation hotspots were evaluated in these patients using MALDI-TOF mass spectrometry were analyzed. *PIK3CA* amplifications were analyzed using real-time quantitative PCR. Regarding patients with intestinal-type gastric cancer, those with mutations in PI3K/AKT pathway genes were also more likely to have tumors located in the lower-third of the stomach than were those without mutations. Regarding patients with diffuse-type gastric cancer, those with PI3K/AKT pathway mutations were more likely to have tumors located in the upper-third of the stomach and to have more hematogenous metastases, particularly in the liver and lungs, than were patients without such mutations (22.2% vs. 4.5%). No significant survival difference was observed between patients with vs. without PI3K/AKT pathway mutations. Mutations in PI3K/AKT pathway genes were associated with hematogenous metastasis in patients with diffuse-type gastric cancer. Only when the tumors were located in the middle-third of stomach, tumor with mutations of the *PIK3CA* gene or mutations of the PI3K/AKT pathway genes were associated with more EBV infection than those without mutations. Patients with *PIK3CA* amplifications were more likely to have diffuse-type and poorly differentiated gastric cancers and were more likely to experience peritoneal recurrence compared with those without *PIK3CA* amplifications. Even upon subgroup analysis, *PI3KCA* amplifications were found to not affect the patients’ outcomes.

## INTRODUCTION

Although the incidence of gastric cancer has continued to decline worldwide, it remains the fourth most common cancer and the second most common cause of cancer death worldwide [1]. Approximately 90% recurrence occurs within 3 years after curative surgery for gastric cancer and is generally associated with a poor outcome [2].

It is known that the accumulation of genetic alterations leads to the development of gastric carcinoma [3]. Most of the genetic mutations in gastric carcinoma correlate with changes in biological signals, such as those in the phosphatidylinositol 3-kinase/AKT/mammalian target of the rapamycin pathway (PI3K/AKT/mTOR pathway) [4]. *PI3K* signaling is a crucial regulator of many essential cellular processes, including cell growth, metabolism, survival, metastasis, and resistance to chemotherapy [5]. Although little substantial evidence exists regarding whether the PI3K/AKT pathway is frequently altered in gastric carcinomas, its precise function remains to be determined [5-7]. An understanding of the biological pathways leading to the development of gastric carcinoma will provide an opportunity to improve targeted therapies.

It was reported that *PI3K* and *AKT* proteins are significantly overexpressed in tumor tissues and in tumors with lymph node metastasis [8]. Furthermore, the simultaneous expression of *PI3K*, *p-AKT*, and *p-mTOR* was an independent prognostic factor of a poor outcome [9]. Therefore, investigation of aspects of the PI3K/AKT/mTOR pathway in gastric cancers may yield useful biomarkers and lead to the development of targeted therapeutic agents for these cancers [10].

*PIK3CA* mutations and amplifications are two major causes of the overactivation of the PI3K/AKT pathway in gastric cancers [11]. Deregulation of the PI3K/AKT pathway can occur subsequent to the existence of oncogenic mutations in the *PIK3CA* gene [12]. The *PIK3CA* gene, which encodes the catalytic subunit pf *PI3K*, is mutated at a high frequency in gastric cancer cell lines and tumor tissues [13]. Several studies have demonstrated that somatic mutations in *PI3KCA* are present in 4%-25% of patients with gastric carcinoma [14-18]. However, there was no association between *PIK3CA* mutations and clinicopathological features. The correlation between *PIK3CA* mutations and EBV infection remains controversial; some authors [19] reported a strong correlation, but other authors [20] reported no significant correlation between them. As previously reported [19], EBV-infected gastric cancer was associated with *PIK3CA* mutation, particularly those occurring in the body of the stomach.

Earlier studies reported a higher frequency of *PIK3CA* amplifications than mutations [11, 21-23]. The frequency of *PI3KCA* amplifications has been reported to be 13%-67% [11, 21-23]. Some studies [11, 21] demonstrated that *PIK3CA* amplifications were associated with a poor prognosis for gastric cancer patients; however, others reported no association with their clinicopathological features [22, 23]. Therefore, whether *PIK3CA* amplifications can serve as a prognostic indicator for gastric cancers remains unclear.

In this study, we examined the effects of the mutation spectra of the PI3K/AKT pathway genes in a Chinese population. However, whole-genome sequencing is a time-consuming and expensive analytical method. Therefore, a high-throughput assay is a crucial tool for clinical practices. Based on the previous results of a whole-genome sequencing investigation and the development of MALDI-TOF mass spectrometry for nucleic-acid analysis, we designed a 39 hot-spot Sequenom MassARRAY platform. We used this platform to analyze mutation spectra and molecular features of the PI3K/AKT pathway and used real-time quantitative PCR (qPCR) to investigate *PIK3CA* amplifications in patients with gastric cancer and the associations with clinicopathological characteristics, recurrence patterns and prognoses.

## RESULTS

### Clinicopathological characteristics

#### Mutations in PI3K/AKT pathway genes

**Figure 1:**
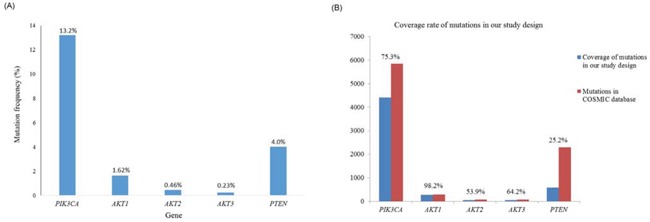
**A.** The frequency of the selected mutation hot spots in five genes within the PI3K/AKT pathway according to MassARRAY. **B.** The coverage rate of the selected mutation hot spots in five genes within the PI3K/AKT pathway according to MassARRAY compared with the COSMIC database.

PI3K/AKT pathway genetic mutations were found in 69 (16%) of the 431 patients. As shown in Figure 1(A), the most commonly mutated genes were *PIK3CA* (13.2%), followed by *PTEN* (4.0%), *AKT3* (1.62%), *AKT2* (0.46%), and *AKT1* (0.23%).

As shown in Supplementary Table 1, the most prevalent hotspot mutations in the *PIK3CA* gene were c.1633G>A (n=14), followed by c.3140A>G (n=11) and c.1624G>A (n=10), and then by the other mutations shown in the table. The most prevalent hotspot mutations in the *PTEN* gene were c.1003C>T (n=5) and c.800delA (n=5), followed by c.518G>A (n=3), and then by the other mutations shown in the table. The most prevalent hotspot mutations in the *AKT3* gene were c.49G>A (n=4), followed by c.496C>T (n=2). There were two patients with hotspot mutations in *AKT2*; one of whom had the c.1063C>T mutation and the other of whom had the c.904A>G mutation. There was only one patient with the c.49G>A hotspot mutation in the *AKT1* gene. All of the hotspot mutations selected for this study were non-silent mutations resulting in an amino acid change, a stop codon or frame shift.

There were 11 patients with two hotspot mutations, whereas no patient with more than two hotspot mutations. The general data of the 11 patients were shown in Supplementary Table 2. Among the 11 patients, four patients have two hotspot mutations in the same gene, including two patients with two hotspot mutations in *PTEN* gene and two patients with two hotspot mutations in *PIK3CA* gene. The other seven patients have two hotspot mutations in different genes, including four patients with *PIK3CA* and *PTEN* mutations, two patients with *PTEN* and *AKT3* mutations, and one patient with *PIK3CA* and *AKT3* mutations. Among the 11 patients, seven patients had tumor recurrence and one of them received another surgery for regional lymph node recurrence and was alive more than 20 years after the second operation. The other six patients with tumor recurrence all died of cancer. Four out of the 11 patients had no tumor recurrence and one of them died of second primary cancer as hepatoma. Three patients were alive without tumor recurrence until the last follow-up.

Among the 11 patients, two patients had simultaneous EBV infection and *PIK3CA* mutations and both of them had stage III gastric cancer and had tumor recurrence within one year after surgery. Both of them died of tumor recurrence.

The correlation between the clinicopathological characteristics and the mutation status of the PI3K/AKT pathway genes is shown in Table 1. Of the 69 patients with at least one mutation in the PI3K/AKT pathway genes, 51 had intestinal-type gastric cancer (73.9%), which was significantly more frequent than in those without any mutations in the PI3K/AKT pathway genes (184/362, 50.8%, *P*=0.001). No significant difference was observed between these two groups with respect to other clinicopathological characteristics.

**Table 1:** Clinical profile in all gastric cancer patients

Variables	PI3K/AKT pathway mutation (−) n=362 n (%)	PI3K/AKT pathway mutation (+) n=69 n (%)	*P* value	*PIK3CA* amplification (−) n=225 n (%)	*PIK3CA* amplification (+) n=206 n (%)	*P* value
Age			1.000			0.493
<65yrs	147 (40.6)	28 (40.6)		95 (42.2)	80 (38.8)	
≥65yrs	215 (59.4)	41 (59.4)		130 (57.8)	126 (61.2)	
Gender			0.389			0.057
Male	252 (69.6)	52 (75.4)		168 (74.7)	136 (66)	
Female	110 (30.4)	17 (24.6)		57 (25.3)	70 (34)	
Tumor size			0.140			0.552
<5cm	145 (40.1)	21 (30.4)		90 (40)	76 (36.9)	
≥5cm	217 (59.9)	48 (69.6)		135 (60)	130 (63.1)	
Tumor location			0.750			0.774
Upper stomach	69 (19.1)	11 (15.9)		45 (20)	35 (17)	
Middle stomach	118 (32.6)	23 (33.3)		70 (31.1)	71 (34.5)	
Lower stomach	163 (45.0)	34 (49.3)		104 (46.2)	93 (45.1)	
Whole stomach	12 (3.3)	1 (1.4)		6 (2.7)	7 (3.4)	
Cell differentiation			0.122			0.031
Poor	204 (56.4)	30 (43.5)		109 (48.4)	125 (60.7)	
Moderate	151 (41.7)	38 (55.1)		113 (50.2)	76 (36.9)	
Well	7 (1.9)	1 (1.4)		3 (1.3)	5 (2.4)	
Gross appearance			0.157			0.477
Superficial type	49 (13.5)	7 (10.1)		30 (13.3)	26 (12.6)	
Borrmann type 1&2	106 (29.3)	14 (20.3)		57 (25.3)	63 (30.6)	
Borrmann type 3&4	207 (57.2)	48 (69.6)		138 (61.3)	117 (56.8)	
Stromal reaction type			0.673			0.138
Medullary type	56 (15.5)	9 (13.0)		40 (17.8)	25 (12.1)	
Intermediate type	189 (52.2)	40 (58.0)		121 (53.8)	108 (52.4)	
Scirrhous type	117 (32.3)	20 (29.0)		64 (28.4)	73 (35.4)	
Lauren's histology			0.001			0.004
Intestinal type	184 (50.8)	51 (73.9)		138 (61.3)	97 (47.1)	
Diffuse type	178 (49.2)	18 (26.1)		87 (38.7)	109 (52.9)	
Lymphovascular invasion			0.885			0.915
Absent	102 (28.2)	20 (29.0)		63 (28)	59 (28.6)	
Present	260 (71.8)	49 (71.0)		162 (72)	147 (71.4)	
Pathological T category			0.322			0.550
T1	61 (16.9)	6 (8.7)		32 (14.2)	35 (17)	
T2	58 (16.0)	11 (15.9)		41 (18.2)	28 (13.6)	
T3	124 (34.3)	24 (34.8)		75 (33.3)	73 (35.4)	
T4	119 (32.7)	28 (40.6)		77 (34.2)	70 (34)	
Pathological N category			0.843			0.490
N0	115 (31.8)	24 (34.8)		71 (31.6)	68 (33)	
N1	58 (16.0)	13 (18.8)		42 (18.7)	29 (14.1)	
N2	94 (26.0)	16 (23.2)		59 (26.2)	51 (24.8)	
N3	95 (26.2)	16 (23.2)		53 (23.6)	58 (28.2)	
Pathological TNM Stage			0.762			0.591
I	77 (21.3)	12 (17.4)		47 (20.9)	42 (20.4)	
II	102 (28.2)	20 (29.0)		68 (30.2)	54 (26.2)	
III	183 (50.6)	37 (53.6)		110 (48.9)	110 (53.4)	

Because the biological behaviors of diffuse-type and intestinal-type gastric cancers differ, we separated the intestinal-type and diffuse-type gastric cancer cases when analyzing the differences between patients with and without mutations in PI3K/AKT pathway genes.

Among the 431 patients in this study, 235 of them (54.5%) had an intestinal-type gastric cancer and 196 of them (45.5%) had a diffuse-type gastric cancer. Regarding intestinal-type gastric cancer, patients with PI3K/AKT pathway mutations were more likely to have tumors in the lower-third of the stomach (Table 2). Regarding diffuse-type gastric cancer, patients with mutations in the PI3K/AKT pathway were more likely to have tumors in the upper-third of the stomach (Table 3).

**Table 2:** Clinical profile in intestinal-type gastric cancer patients

Variables	PI3K/AKT pathway mutation (−) n=184 n(%)	PI3K/AKT pathway mutation (+) n=51 n(%)	*P* value	*PIK3CA* amplification (−) n=138 n (%)	*PIK3CA* amplification (+) n=97 n (%)	*P* value
Age			0.736			0.673
<65yrs	59 (32.1)	18 (35.3)		47 (34.1)	30 (30.9)	
≥65yrs	125 (67.9)	33 (64.7)		91 (65.9)	67 (69.1)	
Gender			1.000			0.746
Male	145 (78.8)	40 (78.4)		110 (79.7)	75 (77.3)	
Female	39 (21.2)	11 (21.6)		28 (20.3)	22 (22.7)	
Tumor size			0.200			0.503
<5cm	82 (44.6)	17 (33.3)		61 (44.2)	38 (39.2)	
≥5cm	102 (55.4)	34 (66.7)		77 (55.8)	59 (60.8)	
Tumor location			0.028			0.302
Upper stomach	42 (22.8)	4 (7.8)		24 (17.4)	22 (22.7)	
Middle stomach	51 (27.7)	16 (31.4)		39 (28.3)	28 (28.9)	
Lower stomach	90 (48.9)	30 (58.8)		74 (53.6)	46 (47.4)	
Whole stomach	1 (0.5)	1 (2.0)		1 (0.7)	1 (1)	
Cell differentiation			0.451			0.744
Poor	33 (17.9)	13 (25.5)		26 (18.8)	20 (20.6)	
Moderate	145 (78.8)	37 (72.5)		110 (79.7)	72 (74.2)	
Well	6 (3.3)	1 (2.0)		2 (1.4)	5 (5.2)	
Gross appearance			0.115			0.035
Superficial type	24 (13.0)	6 (11.8)		22 (15.9)	8 (8.2)	
Borrmann type 1&2	66 (35.9)	11 (21.6)		37 (26.8)	40 (41.2)	
Borrmann type 3&4	94 (51.1)	34 (66.7)		79 (57.2)	49 (50.5)	
Stromal reaction type			0.249			0.171
Medullary type	21 (11.4)	7 (13.7)		21 (15.2)	7 (7.2)	
Intermediate type	133 (72.3)	31 (60.8)		92 (66.7)	72 (74.2)	
Scirrhous type	30 (16.3)	13 (25.5)		25 (18.1)	18 (18.6)	
Lymphovascular invasion			0.596			0.883
Absent	49 (26.6)	16 (31.4)		39 (28.3)	26 (26.8)	
Present	135 (73.4)	35 (68.6)		99 (71.7)	71 (73.2)	
Pathological T category			0.368			0.693
T1	33 (17.9)	5 (9.8)		20 (14.5)	18 (18.6)	
T2	39 (12.2)	9 (17.6)		30 (21.7)	18 (18.6)	
T3	58 (31.5)	17 (33.3)		42 (30.4)	33 (34)	
T4	54 (29.3)	20 (39.2)		46 (33.3)	28 (28.9)	
Pathological N category			0.861			0.717
N0	71 (38.6)	21 (41.2)		56 (40.6)	36 (37.1)	
N1	29 (15.8)	8 (15.7)		22 (15.9)	15 (15.5)	
N2	50 (27.2)	11 (21.6)		37 (26.8)	24 (24.7)	
N3	34 (18.5)	11 (21.6)		23 (16.7)	22 (22.7)	
Pathological TNM Stage			0.659			0.877
I	49 (26.6)	11 (21.6)		35 (25.4)	25 (25.8)	
II	57 (31.0)	15 (29.4)		44 (31.9)	28 (28.9)	
III	78 (42.4)	25 (49.0)		59 (42.8)	44 (45.4)	

**Table 3:** Clinical profile in diffuse-type gastric cancer patients

Variables	PI3K/AKT pathway mutation (−) n=178 n (%)	PI3K/AKT pathway mutation (+) n=18 n (%)	*P* value	*PIK3CA* amplification (−) n=87 n (%)	*PIK3CA* amplification(+) n=109 n (%)	*P* value
Age			0.805			0.250
<65yrs	88 (49.4)	10 (55.6)		48 (55.2)	50 (45.9)	
≥65yrs	90 (50.6)	8 (44.4)		39 (44.8)	59 (54.1)	
Gender			0.801			0.143
Male	107 (60.1)	12 (66.7)		58 (66.7)	61 (56)	
Female	71 (39.9)	6 (33.3)		29 (33.3)	48 (44)	
Tumor size			0.308			0.880
<5cm	63 (35.4)	4 (22.2)		29 (33.3)	38 (34.9)	
≥5cm	115 (64.6)	14 (77.8)		58 (66.7)	71 (65.1)	
Tumor location			0.007			0.087
Upper third stomach	27 (15.2)	7 (38.9)		21 (24.1)	13 (11.9)	
Middle third stomach	67 (37.6)	7 (38.9)		31 (35.6)	43 (39.4)	
Lower third stomach	73 (41.0)	4 (22.2)		30 (34.5)	47 (43.1)	
Whole stomach	11 (6.2)	0		5 (5.7)	6 (5.5)	
Cell differentiation			0.850			0.535
Poor	171 (96.1)	17 (94.4)		83 (95.4)	105 (96.3)	
Moderate	6 (3.4)	1 (5.6)		3 (3.4)	4 (3.7)	
Well	1 (0.6)	0		1 (1.1)	0	
Gross appearance			0.437			0.324
Superficial type	25 (14.0)	1 (5.6)		8 (9.2)	18 (16.5)	
Borrmann type 1&2	40 (22.5)	3 (16.7)		20 (23)	23 (21.1)	
Borrmann type 3&4	113 (63.5)	14 (77.8)		59 (67.8)	68 (62.4)	
Stromal reaction type			0.263			0.592
Medullary type	35 (19.7)	2 (11.1)		19 (21.8)	18 (16.5)	
Intermediate type	56 (31.5)	9 (50.0)		29 (33.3)	36 (33)	
Scirrhous type	87 (48.9)	7 (38.9)		39 (44.8)	55 (50.5)	
Lymphovascular invasion			0.596			0.752
Absent	53 (29.8)	4 (22.2)		24 (27.6)	33 (30.3)	
Present	125 (70.2)	14 (77.8)		63 (72.4)	76 (69.7)	
Pathological T category			0.697			0.855
T1	28 (15.7)	1 (5.6)		12 (13.8)	17 (15.6)	
T2	19 (10.7)	2 (11.1)		11 (12.6)	10 (9.2)	
T3	66 (37.1)	7 (38.9)		33 (37.9)	40 (36.7)	
T4	65 (36.5)	8 (44.4)		31 (35.6)	42 (38.5)	
Pathological N category			0.583			0.118
N0	44 (24.7)	3 (16.7)		15 (17.2)	32 (19.4)	
N1	29 (16.3)	5 (27.8)		20 (23)	14 (12.8)	
N2	44 (24.7)	5 (27.8)		22 (25.3)	27 (24.8)	
N3	61 (34.3)	5 (27.8)		30 (34.5)	36 (33)	
Pathological TNM Stage			0.510			0.818
I	28 (15.7)	12 (5.6)		12 (13.8)	17 (15.6)	
II	45 (25.3)	20 (27.8)		24 (27.6)	26 (23.9)	
III	105 (59.0)	37 (66.7)		51 (58.6)	66 (60.6)	

As shown in Table 4, tumors with an EBV infection were more likely to be located in the middle-third of the stomach (42.2%), whereas tumors without an EBV infection were more frequently located in the lower-third of the stomach (50.4%). Moreover, only when the tumors were located in the middle-third of the stomach were tumors with mutations in the *PIK3CA* gene or in the PI3K/AKT pathway genes more likely to harbor EBV infection than were those without such mutations (Table 5).

**Table 4:** Clinical profile in gastric cancer patients with/without EBV infection

Variables	Without EBV infection n=367 n(%)	With EBV infection n=64 n(%)	*P* value
Age			0.171
<65yrs	144 (39.2)	31 (48.4)	
≥65yrs	223 (60.8)	33 (51.6)	
Gender			1.000
Male	259 (70.6)	52 (70.3)	
Female	108 (29.4)	17 (29.7)	
Tumor size			1.000
<5cm	141 (38.4)	25 (39.1)	
≥5cm	226 (61.6)	39 (60.9)	
Tumor location			<0.001
Upper stomach	55 (15)	25 (39.1)	
Middle stomach	114 (31.1)	27 (42.2)	
Lower stomach	185 (50.4)	12 (18.8)	
Whole stomach	13 (3.5)	0	
Cell differentiation			0.122
Poor	199 (56.4)	35 (54.7)	
Moderate	160 (41.7)	29 (45.3)	
Well	8 (1.9)	0	
Gross appearance			0.087
Superficial type	50 (13.6)	6 (9.4)	
Borrmann type 1&2	95 (25.9)	25 (39.4)	
Borrmann type 3&4	222 (60.5)	33 (51.6)	
Stromal reaction type			0.850
Medullary type	54 (14.7)	11 (17.2)	
Intermediate type	195 (53.1)	34 (53.1)	
Scirrhous type	118 (32.2)	19 (29.7)	
Lauren's histology			1.000
Intestinal type	200 (54.5)	35 (54.7)	
Diffuse type	167 (45.5)	29 (45.3)	
Lymphovascular invasion			0.097
Absent	98 (26.7)	24 (37.5)	
Present	269 (73.3)	40 (62.5)	
Pathological T category			0.753
T1	59 (16.1)	8 (12.5)	
T2	57 (15.5)	12 (18.8)	
T3	124 (33.8)	24 (37.5)	
T4	127 (34.6)	20 (31.3)	
Pathological N category			0.239
N0	122 (33.2)	17 (26.6)	
N1	56 (15.3)	15 (23.4)	
N2	97 (26.4)	13 (20.3)	
N3	92 (25.1)	19 (29.7)	
Pathological TNM Stage			0.715
I	78 (21.3)	11 (17.2)	
II	102 (27.8)	20 (31.3)	
III	187 (51.0)	33 (51.6)	

**Table 5:** The association between genetic mutations in the PI3K/AKT pathway, *PIK3CA* amplifications and EBV infection in gastric cancer

	*PIK3CA* gene mutations			*PIK3CA* gene amplifications			PI3K/AKT pathway mutation		
	Without n=127 n (%)	With n=14 n (%)	*P* value	Without n=87 n (%)	With n=109 n (%)	*P* value	Without n=118 n (%)	With n=23 n (%)	*P* value
EBV infection			0.001			0.689			0.046
Absent	108 (85)	6 (42.9)		73 (83.9)	94 (86.2)		99 (83.9)	15 (65.2)	
Present	19 (15)	8 (57.1)		14 (16.1)	15 (13.8)		19 (16.1)	8 (34.8)	

### PIK3CA amplifications

Of the 431 patients in this study, *PIK3CA* amplifications were found in 206 of the patients (47.8%). The mean copy number in these patients was 4.33±1.66, with a range of 0.3-6.02. As previously reported [11], amplification of the *PIK3CA* gene was defined as a copy number of ≥3 with a *P*-value of <0.05. The correlation between the clinicopathological characteristics and the mutation status of the PI3K/AKT pathway genes is shown in Table 1. A greater percentage of the patients with *PIK3CA* amplifications had diffuse-type gastric cancer (52.9% vs. 38.7%, *P*=0.004) and poorly differentiated adenocarcinoma (60.7% vs. 48.4%, *P*=0.031) compared with those patients without *PIK3CA* amplifications. Regarding intestinal-type gastric cancer, patients with mutations in the PI3K/AKT pathway genes were more likely to have a less superficial-type of gastric cancer (Table 2). Regarding diffuse-type gastric cancer, there was no significant difference between the clinicopathological characteristics of patients with or without mutations in the PI3K/AKT pathway genes (Table 3).

As shown in Table 5, there is no correlation between EBV infection and patients with or without *PI3KCA* amplifications.

### Initial recurrence patterns

#### Mutations in PI3K/AKT pathway genes

A total of 154 patients (35.7%) experienced tumor recurrence within a median *period* of 31.7 months (range: 0.47-183.0 months) after the initial follow-up visit. Patients with mutations in the PI3K/AKT pathway genes had more hematogenous metastases than did those without mutations (18.8% vs. 7.5% for patients with vs. without mutations, respectively; *P*=0.006), particularly liver metastasis (18.8% vs. 7.2% for patients with vs. without mutations, respectively; *P*=0.005). Regarding intestinal-type gastric cancer, no significant difference was observed between the recurrence patterns in patients with or without PI3K/AKT pathway gene mutations (Table 6). Regarding diffuse-type gastric cancer, patients with mutations in the PI3K/AKT pathway genes were more likely to have hematogenous metastases than were those without mutations (22.2% vs. 4.5% for patients with vs. without mutations, respectively; *P*=0.016), particularly metastases in the liver and lungs (Table 7).

**Table 6:** The initial recurrence pattern in intestinal-type gastric cancer patients after curative surgery

	PI3K/AKT pathway mutation (−) n=184 n (%)	PI3K/AKT pathway mutation (+) n=51 n (%)	*P* value	*PIK3CA* amplification (−) n=138 n (%)	*PIK3CA* amplification (+) n=97 n (%)	*P* value
Total patients with recurrence	57 (31.0)	22 (43.1)	0.131	46 (33.3)	33 (34)	1.000
Locoregional recurrence	20 (10.9)	11 (21.6)	0.060	20 (14.5)	12 (12.4)	0.702
Perigastric area	5 (2.7)	2 (3.9)	0.647	4 (2.9)	3 (3.1)	1.000
Hepatoduodenal ligament	16 (8.7)	8 (15.7)	0.188	14 (10.1)	10 (10.3)	1.000
Anastomosis	5 (2.7)	2 (3.9)	0.647	5 (3.6)	2 (2.1)	0.703
Abdominal wall	10 (5.4)	4 (7.8)	0.510	9 (6.5)	5 (5.2)	0.783
Duodenal stump	1 (0.5)	0	1.000	1 (0.7)	1 (1)	1.000
Distant metastasis	41 (22.3)	13 (25.5)	0.707	28 (20.3)	27 (27.8)	0.211
Peritoneal dissemination	15 (8.2)	6 (11.8)	0.413	10 (7.2)	11 (11.3)	0.354
Hematogenous metastasis	19 (10.3)	9 (17.6)	0.219	15 (10.9)	13 (13.4)	0.548
Liver	18 (9.8)	9 (17.6)	0.137	15 (10.9)	12 (12.4)	0.836
Lung	6 (3.3)	1 (2.0)	1.000	3 (2.2)	4 (4.1)	0.451
Bone	5 (2.7)	0	0.588	3 (2.2)	2 (2.1)	1.000
Brain	1 (0.5)	0	1.000	0	1 (1)	0.413
Distant lymphatic recurrence	10 (5.4)	4 (7.8)	0.510	10 (7.2)	5 (5.2)	0.597
Virchow's node	2 (1.1)	1 (2.0)	0.522	2 (1.4)	1 (1)	1.000
Para-aortic lymph node	9 (4.9)	3 (5.9)	0.726	8 (5.8)	5 (5.2)	1.000

Some patients had more than one recurrence pattern

**Table 7:** The initial recurrence pattern in diffuse-type gastric cancer patients after curative surgery

	PI3K/AKT pathway mutation (−) n=178 n (%)	PI3K/AKT pathway mutation (+) n=18 n (%)	*P* value	*PIK3CA* amplification (−) n=87 n (%)	*PIK3CA* amplification (+) n=109 n (%)	*P* value
Total patients with recurrence	68 (38.2)	7 (38.9)	1.000	26 (29.9)	49 (45)	0.038
Locoregional recurrence	12 (6.7)	3 (16.7)	0.146	7 (8)	8 (7.3)	1.000
Perigastric area	2 (1.1)	1 (5.6)	0.252	2 (2.3)	1 (0.9)	0.586
Hepatoduodenal ligament	7 (3.9)	3 (16.7)	0.052	4 (4.6)	6 (5.5)	1.000
Anastomosis	6 (3.4)	0	1.000	4 (4.6)	2 (1.8)	0.409
Abdominal wall	4 (2.2)	2 (11.1)	0.096	3 (3.4)	3 (2.8)	1.000
Distant metastasis	45 (25.3)	7 (38.9)	0.257	20 (23)	32 (29.4)	0.334
Peritoneal dissemination	31 (17.4)	4 (22.2)	0.693	12 (13.8)	23 (21.1)	0.196
Hematogenous metastasis	8 (4.5)	4 (22.2)	0.016	6 (6.9)	6 (5.5)	0.769
Liver	8 (4.5)	4 (22.2)	0.016	6 (6.9)	6 (5.5)	0.769
Lung	2 (1.1)	2 (11.1)	0.043	3 (3.4)	1 (0.9)	0.324
Bone	2 (1.1)	1 (5.6)	0.252	2 (2.3)	1 (0.9)	0.586
Brain	1 (0.6)	0	1.000	0	1 (0.9)	1.000
Adrenal	1 (0.6)	0	1.000	0	1 (0.9)	1.000
Skin	1 (0.6)	0	1.000	0	1 (0.9)	1.000
Distant lymphatic recurrence	16 (9.0)	1 (5.6)	1.000	7 (8)	10 (9.2)	1.000
Virchow's node	2 (1.1)	0	1.000	0	2 (1.8)	0.504
Lymphangitic carcinomatosis	1 (0.6)	0	1.000	1 (1.1)	0	0.444
Para-aortic lymph node	14 (7.9)	1 (5.6)	1.000	6 (6.9)	9 (8.3)	0.792

Some patients had more than one recurrence pattern

### PIK3CA amplification

Among the 206 patients with *PIK3CA* amplifications, 34 patients (16.5%) experienced peritoneal recurrence, which was significantly higher than the rate for those patients without *PIK3CA* amplifications (22/225, 9.8%, *P*=0.045). With regard to intestinal-type or diffuse-type gastric cancer, however, there was no significant difference between the initial recurrence patterns in patients with or without *PIK3CA* amplifications.

### Disease-free survival

The 5-year disease-free survival rates of patients with or without mutations in the PI3K/AKT pathway genes were not significantly different (35.9% vs. 47.2% for patients with vs. without mutations, respectively; *P*=0.156), and those of the two groups with either early gastric cancer (T1 tumors, 80% vs. 83.6%, *P*=0.800) or advanced gastric cancer (T2-T4 tumors, 31.0% vs. 40.1%, *P*=0.235) were also not significantly different.

The 5-year disease-free survival rates of patients with or without *PIK3CA* amplifications were similar (44.1% vs. 46.7%, *P*=0.367), and those of the two groups with either early gastric cancer (79.6% vs. 88.6%, *P*=0.588) or advanced gastric cancer (37% vs. 40.2%, *P*=0.327) were also note significantly different.

Univariate analysis showed that gender, tumor size, tumor location, gross appearance, stromal reaction type, and the pathological T and N categories were prognostic factors for disease-free survival (Table 8). Multivariate analysis showed that tumor size, tumor location, gross appearance, and pathological T and N categories were independent prognostic factors that affected disease-free survival (Table 9).

**Table 8:** Univariate analysis of factors affecting survival of gastric cancer patients after curative surgery by the Kaplan–Meier method

	Disease-free survival			Overall survival		
	HR	95%CI	*P* value	HR	95%CI	*P* value
Age (y/o)			0.061			0.001
<65	1.00			1.00		
≥65	1.30	0.988-1.703		1.65	1.229-2.206	
Gender			0.037			0.002
Male	1.00			1.00		
Female	0.72	0.534-0.981		0.59	0.425-0.818	
Tumor size (cm)			<0.001			<0.001
<5	1.00			1.00		
≥5	2.52	1.859-3.417		2.54	1.839-3.499	
Tumor location			<0.001			<0.001
Upper third stomach	1.00			1.00		
Middle third stomach	0.60	0.408-0.876		0.59	0.396-0.890	
Lower third stomach	0.76	0.539-1.064		0.84	0.595-1.193	
Whole stomach	2.38	1.196-4.746		2.46	1.238-4.902	
Gross appearance			<0.001			<0.001
Superficial type	1.00			1.00		
Borrmann type 1&2	2.53	1.426-4.503		2.67	1.471-4.861	
Borrmann type 3&4	4.56	2.678-7.769		4.27	2.466-7.406	
Cell differentiation			0.333			0.368
Poor	1.00			1.00		
Moderate	0.82	0.632-1.066		0.84	0.636-1.099	
Well	0.98	0.362-2.656		0.68	0.252-1.855	
Lauren's classification			0.484			0.738
Intestinal type	1.00			1.00		
Diffuse type	1.10	0.847-1.418		1.05	0.798-1.374	
Stromal Reaction type			0.003			0.012
Medullary type	1.00			1.00		
Intermediate type	1.59	1.042-2.437		1.61	1.048-2.472	
Scirrhous type	2.11	1.358-3.280		1.96	1.256-3.065	
Pathological T category			<0.001			<0.001
T1	1.00			1.00		
T2	1.87	1.019-3.431		1.89	1.008-3.538	
T3	2.83	1.666-4.819		2.62	1.504-4.560	
T4	5.24	3.145-8.727		5.05	2.990-8.537	
Pathological N category			<0.001			<0.001
N0	1.00			1.00		
N1	1.14	0.725-1.799		1.08	0.666-1.743	
N2	2.38	1.653-3.421		2.37	1.632-3.446	
N3	4.84	3.369-6.940		5.56	3.814-8.113	
PI3K/AKT pathway mutation			0.156			0.529
Absent	1.00			1.00		
Present	1.27	0.913-1.770		0.89	0.625-1.273	
*PIK3CA* amplification			0.103			0.368
Absent	1.00			1.00		
Present	1.25	0.956-1.634		1.13	0.871-1.453	

**Table 9:** Multivariate Cox proportional-hazards model using the forward logistics regression stepwise procedure for the analysis of the survival of the gastric cancer patients after curative surgery

	Disease-free survival			Overall survival		
	HR	95%CI	*P* value	HR	95%CI	*P* value
Age (y/o)						0.001
<65				1.00		
≥65				1.78	1.318-2.393	
Tumor size (cm)			0.013			
<5	1.00					
≥5	1.54	1.093-2.155				
Tumor location			0.039			
Upper third stomach	1.00					
Middle third stomach	0.68	0.459-1.013				
Lower third stomach	0.63	0.439-0.889				
Whole stomach	1.05	0.515-2.137				
Gross appearance			0.006			0.007
Superficial type	1.00			1.00		
Borrmann type 1&2	1.55	0.782-3.073		1.67	0.829-3.368	
Borrmann type 3&4	2.36	1.199-4.628		2.47	1.244-4.919	
Pathological T category			0.044			<0.001
T1	1.00			1.00		
T2	1.10	0.551-2.213		0.82	0.396-1.698	
T3	1.15	0.584-2.258		0.98	0.487-1.978	
T4	1.71	0.857-3.392		1.71	0.867-3.378	
Pathological N category			<0.001			<0.001
N0	1.00			1.00		
N1	0.83	0.518-1.321		0.89	0.543-1.467	
N2	1.48	0.997-2.187		1.71	1.150-2.556	
N3	2.69	1.797-4.019		4.15	2.758-6.242	

For patients with intestinal-type gastric cancer, the 5-year disease-free survival rates of those with or without mutations in the PI3K/AKT pathway genes were not significantly different (36.9% vs. 53.7% for those with vs. without mutations, respectively; *P*=0.051). For patients with diffuse-type gastric cancer, the 5-year disease-free survival rates of those with or without mutations in the PI3K/AKT pathway genes were not significantly different (30.0% vs. 40.3% for patients with vs. without mutations, respectively; *P*=0.897).

For patients with intestinal-type gastric cancer, the 5-year disease-free survival rates of those with or without *PIK3CA* amplifications were not significantly different (49.8% vs. 50.4% for those with vs. without mutations, respectively; *P*=0.533). For patients with diffuse-type gastric cancer, the 5-year disease-free survival rates of those with or without *PIK3CA* amplifications were not significantly different (38.9% vs. 38.5% for patients with vs. without mutations, respectively; *P*=0.573).

### Overall survival

#### Mutations in PI3K/AKT pathway genes

The 5-year overall survival rates of patients with or without mutations in the PI3K/AKT pathway genes were also not significantly different (55.5% vs. 56.4% *P*=0.529), and those of the two groups of either early gastric cancer (83.3% vs. 88.6%, *P*=0.977) or advanced gastric cancer (51.4% vs. 49.2%, *P*=0.303) were also not significantly different.

The 5-year overall survival rates of patients with or without *PIK3CA* amplifications were not significantly different (54.4% vs. 57.1%, *P*=0.102), and those of the two groups with either early gastric cancer (85.4% vs. 91.3%, *P*=0.662) or advanced gastric cancer were also not significantly different (47.3% vs. 51.5%, *P*=0.055).

Univariate analysis showed that age, gender, tumor size, tumor location, gross appearance, stromal reaction type, and the pathological T category and N category were prognostic factors for overall survival (Table 8). Multivariate analysis showed that age, gross appearance, and the pathological T and N categories were independent prognostic factors that affected overall survival (Table 9).

Regarding patients with intestinal-type gastric cancer, the 5-year overall survival rates of patients with or without mutations in the PI3K/AKT pathway genes were not significantly different (57.7% vs. 55.5% for patients with vs. without mutations, respectively; *P*=0.851). For those with diffuse-type gastric cancer, the 5-year overall survival rates of patients with or without mutations in the PI3K/AKT pathway genes were not significantly different (55.5% vs. 53.6% for patients with vs. without mutations, respectively; *P*=0.440).

Regarding patients with intestinal-type gastric cancer, the 5-year overall survival rates of those with or without *PIK3CA* amplifications were not significantly different (54.3% vs. 58.8% for patients with vs. without mutations, respectively; *P*=0.103). Regarding patients with diffuse-type gastric cancer, the 5-year overall survival rates of those with or without *PIK3CA* amplifications were not significantly different (54.5% vs. 54.1% for patients with vs. without mutations, respectively; *P*=0.542).

## DISCUSSION

This study presents the genetic profiles of PI3K/AKT pathway components and the association of genetic mutations in this pathway with the clinicopathological characteristics, initial recurrence patterns and prognoses of gastric cancer patients. Our novel findings showed that intestinal-type gastric cancer with *PI3K/AKT* pathway mutations was associated with tumors in the lower-third of the stomach, whereas diffuse-type gastric cancer with PI3K/AKT pathway mutations was associated with tumors in the upper-third of the stomach; diffuse-type gastric cancer with PI3K/AKT pathway mutations was also associated with more hematogenous metastasis. Mutations in PI3K/AKT pathway genes were not independent risk factors for the overall survival or disease-free survival of either intestinal-type and diffuse-type gastric cancer patients.

As previously reported [19], the authors proposed a molecular classification dividing gastric cancer into four subtypes, including EBV positive, chromosomal instability, microsatellite instability, and genomically stable. Chromosomal instability (including intestinal histology, TP53 mutation, RTK-RAS activation) was more frequently located in the fundus & body (38.8%), followed by the antrum (33.3%) and cardia (25.2%); enrichment of the diffuse histology was observed in the genomically stable group which was more frequently located in the antrum (48.3%), followed by fundus & body (31.0%) and cardia (19.0%). A previous study [24] showed that the PI3K/AKT pathway plays an important role in the development of diffuse-type gastric cancer in the upper stomach and the esophagogastric junction, which is consistent with our findings. Consequently, a survey of PI3K/AKT pathway mutations is required for patients with a diffuse-type gastric cancer in the upper stomach and might be helpful for choosing a targeted therapy. Furthermore, our novel findings revealed that in the case of intestinal-type gastric cancer, patients with PI3K/AKT pathway mutations were more likely to have tumors located in the lower-third of the stomach. It is interesting that the distribution of gastric cancer was correlated with the histological type and with the presence of PI3K/AKT pathway mutations. More patients enrolled in the future are required to investigate these interesting findings.

A previous study [25] revealed that blockade of the PI3K/AKT pathway could inhibit liver metastasis of colorectal cancer. An *in vivo* study [26] showed that inhibition of the PI3K/AKT pathway could decrease the progression of liver metastases of pancreatic neuroendocrine tumors. MiR-7 was reported to function as a tumor suppressor and plays a substantial role in inhibiting the tumorigenesis and reversing the metastasis of hepatocellular carcinoma through the PI3K/AKT/mTOR-signaling pathway both *in vitro* and *in vivo* [27]. Hence, the level of PI3K/AKT pathway activity might be correlated with the hematogenous metastasis of cancers. However, a correlation between PI3K/AKT pathway mutations and the distant metastasis of gastric cancer has not yet been reported. Our novel findings showed that patients with diffuse-type gastric cancer (but not other types of cancers) and PI3K/AKT pathway mutations were more likely to have liver and lung metastases compared with those without such mutations. Therefore, for patients with diffuse-type gastric cancer with liver metastasis, a survey of PI3K/AKT pathway mutations is recommended because targeted therapy might be helpful for patients with PI3K/AKT pathway mutations.

In this study, the mutation rate of *PIK3CA* was 13.2% and that of *PTEN* was 4.0%, which is in accordance with the reported mutation rate of *PIK3CA* in gastric cancer [14] but is lower than the mutation rates (18.75%) observed for *PTEN* in the study of Wen et. al. [28], who performed direct sequencing of all 9 exons of *PTEN*. Although the coverage rate of our MassARRAY for *PIK3CA* mutations was 75.3% that of the COSMIC database, the coverage rate for *PTEN* mutations was only 25.2% (Figure 1). Variations in the detection methods used and in the patient populations may contribute to the lower incidence of *PTEN* mutations observed in the present study.

Some previous reports have suggested that patients with breast cancer who have *PIK3CA* mutations, have a better prognosis than others [29], whereas other authors have suggested that *PIK3CA* mutations are associated with a worse prognosis in colorectal cancer, endometrial cancer and lung cancer patients [30–32]. No reports investigating the correlation between PI3K/AKT pathway mutations and patient survival after curative resection of gastric cancer have been published. Only Takahashi et al. [18] reported that *PIK3CA* mutations were not associated with the prognosis of patients with metastatic gastric cancer who were being treated with systemic chemotherapy. In their study, only *PIK3CA* mutations (rather than PI3K/AKT pathway mutations) were examined, and the incidence of *PIK3CA* mutations in gastric cancer patients was only 9 of 163 patients (5.5%). To date, the current study is the only to investigate the effect of mutations in PI3K/AKT pathway genes on the survival of a large population of patients with gastric cancer after curative surgery. Although no significant difference was observed in terms of the disease-free survival and overall survival of patients with or without PI3K/AKT pathway mutations, our results might help guide future studies of the PI3K/AKT pathway in gastric cancer.

Our results showed that only when the tumors were located in the middle-third of the stomach were more tumors with mutations in the *PIK3CA* gene or mutations in PI3K/AKT pathway genes were associated with an EBV infection than were those without such mutations. It is interesting that the correlation between mutations and EBV infection was observed only in the middle-third of the stomach, but not in the upper-third or lower-third of the stomach. The previous study [19] demonstrated that EBV-infected gastric cancer was associated with *PIK3CA* mutation, particularly those occurring in the body of the stomach, which was similar to our findings. There are two possible reasons for the above findings. The first reason is that the EBV infection is higher in the middle-third of the stomach, causing the correlation between EBV infection and *PIK3CA* mutations to be higher in this location of stomach. The second reason is that an EBV infection and *PIK3CA* mutations are more likely to induce carcinogenesis in the middle-third of the stomach. Our results showed that tumors with *PIK3CA* mutations were more likely to be located in the lower-third of the stomach, whereas EBV infections were more frequent in the middle-third of the stomach. Therefore, the second reason might more likely explain our findings. However, various confounding factors, such as selection bias, racial differences, the sex ratio, and the complicated pathways involved in gastric carcinogenesis might have affected the results. Future large-scale studies investigating the correlation between EBV infection, *PIK3CA* mutations and tumor locations are required to address these interesting findings.

*The* Helicobacter pylori (*H. pylori*) VacA toxin was reported to activate the PI3K/AKT pathway, resulting in the phosphorylation and the inhibition of GSK3β activity and the subsequent translocation of β-catenin to the nucleus, which is consistent with the effects of VacA on β-catenin-regulated transcriptional activity [33]. Thus, the PI3K/AKT pathway might play a role in pathogenesis resulting from an *H. pylori* infection, including gastric carcinogenesis. Whether an *H. pylori* infection is associated with mutations in PI3K/AKT pathway genes is unknown. Furthermore, information regarding an *H. pylori* infection is not available for all of our patients. Our future study will investigate the correlation between an *H. pylori* infection and mutations in PI3K/AKT pathway genes.

Regarding *PIK3CA* amplifications in gastric cancer, our results showed that patients with *PIK3CA* amplifications were more likely to have diffuse-type, poorly differentiated gastric cancer and peritoneal seeding compared with patients without *PIK3CA* amplifications. However, the 5-year overall survival and disease-free survival of patients with or without *PIK3CA* amplifications were not significantly different. Regarding intestinal-type or diffuse-type gastric cancer, *PIK3CA* amplifications was not associated with the initial recurrence pattern or with survival. Whether *PIK3CA* amplifications are associated with the prognosis of gastric cancer remains controversial. In the patient series that Shi et al. investigated [11], patients with *PIK3CA* amplifications were found to have with a worse prognosis were than those without *PIK3CA* amplifications; however, Lee et al. [22] reported that *PIK3CA* amplifications were not associated with a worse prognosis. Lee et al. [22] proposed that the possible reason for this discrepancy might be the different proportions of patients with early gastric cancer (more than 40% of the patients in their series compared with only 14% of the patients in the series of Shi et al. [11]). However, the percentage of patients with early gastric cancer in our series was 15.5%, which was similar to that in the series of Shi et al. Our data demonstrated no significantly difference in survival of patients with or without *PIK3CA* amplifications. Additionally, in the present study, no difference in the survival of patients with or without *PIK3CA* amplifications who had early or advanced gastric cancer was found. Consequently, the difference between the survival observed in the study of Shi et al. and that of Lee et al. is unlikely to be due to a difference in the percentage of patients with early gastric cancer. The reliability of clinical data is highly important for this type of study. Various confounding factors might affect the results. In our hospital, the gastric cancer patients were operated upon by experienced surgeons and the pathological review was performed by only one pathologist. Because our hospital is one of the best medical centers in northern Taiwan and more than 60% of our patients are veterans, more than 90% of our patients received postoperative treatment and follow-up in our hospital. We also created the only gastric cancer patient care group in Taiwan more than 20 years ago. We believe that our results are reliable. Based on our data, we conclude that *PIK3CA* amplifications are not associated with the prognosis of gastric cancer.

One of our novel findings is that *PIK3CA* amplifications were associated with more peritoneal recurrences of gastric cancers. This result is interesting and has not been previously reported. Further *in vivo* and intro studies investigating the possible mechanisms underlying the occurrence are required to substantiate our findings. Moreover, physicians should be alert to peritoneal recurrence during the follow-up period, particularly in gastric cancer patients with *PIK3CA* amplifications.

An inverse correlation between *PIK3CA* mutations and *PIK3CA* amplifications was reported in various cancers, including gastric cancer [11, 34, 35]. In our study, among those patients with *PIK3CA* mutations, none had *PIK3CA* amplifications and there were no *PIK3CA* mutations in the patients with *PIK3CA* amplifications. These results suggest that different pathways are involved in *PIK3CA* mutations and amplifications in gastric cancer.

As shown in Figure 1(B), based on comparing the MassARRAY data with that om the COSMIC database, the coverage rate of the selected mutation hotspots in five genes analyzed that are involved in the PI3K/AKT pathway was 75.3% for *PIK3CA*, 98.2% for *AKT1*, 53.9% for *AKT2*, 64.2% for *AKT3*, and 25.2% for *PTEN*. However, not all of the reported somatic alterations, e.g., point mutations, small insertions and deletions, large insertions and deletions, and gene fusion mutations in these genes were examined in this study. The most frequent mutations determined using the COSMIC database and found in previous studies provide a possible target list for investigating their clinical impact via translational genomics using a large sample size, such as that used in this study. Using multiple technologies and multiple types of materials (DNA, RNA, miRNA and protein) is an ideal strategy for comprehensively investigating the pathogenesis of gastric cancer; however, the purpose of the present study was to identify frequent clinically actionable DNA mutations to analyze in clinical samples obtained in the future. In addition, we considered each individual mutation site but not all of the mutations in each of the selected gene, so that sufficient data regarding only the mutation hotspots was obtained using the clinical samples and might have statistical significances.

A limitation of this study was that the mutation screening was limited to certain hotspots within five notable genes involved in the PI3K/AKT downstream pathway, which may have limited the power to detect significant clinicopathological and molecular correlations. In addition, we examined genetic mutations in our study rather than the loss of expression of the relevant encoded proteins, which might have led to underestimation of the actual frequency of genetic inactivation. Furthermore, not all of the patients with gastric cancer who had undergone surgery in our hospital were enrolled in this study. Therefore, selection bias might have occurred. The number of patients with diffuse-type gastric cancer with hematogenous metastasis was limited. Whereas the conclusions drawn might not be reliable, the statistical value of the data obtained is considerable. Nevertheless, our cohort is the largest to date in which the clinicpathological characteristics and recurrence patterns regarding mutations in PI3K/AKT pathway genes and *PIK3CA* amplifications in gastric cancers were investigated. More patients must be enrolled in the future to verify our current conclusions.

In conclusion, our findings provide evidence that mutations in PI3K/AKT pathway-related genes in diffuse-type gastric cancers can affect the patterns of recurrence. Gastric cancer patients with *PIK3CA* amplifications had more diffuse-type and poorly differentiated gastric cancer and more peritoneal recurrences compared with patients without *PIK3CA* amplifications. Physicians should be aware of the possibility of hematogenous metastasis during the follow-up of patients with gastric cancer who have PI3K/AKT pathway mutations, particularly those with diffuse-type gastric cancer. During the follow-up of gastric cancer patients with *PIK3CA* amplifications, physicians should be aware of the possibility of peritoneal recurrence.

## MATERIALS AND METHODS

Between October 2000 and March 2007, 431 patients with gastric cancer who had undergone curative resection were enrolled in this study, which was approved by the Institutional Review Board of the Taipei Veterans General Hospital. Informed consent was obtained and the procedures followed were in accordance with the ethical standards of the committee overseeing human experimentation at the Taipei Veterans General Hospital and the Helsinki Declaration of 1975, as revised in 1983. Patients with a history of gastric surgery or a pathological diagnosis other than adenocarcinoma were excluded. Patients who experienced major surgical complications or surgical mortality were also excluded.

The pathological staging of the gastric cancers was performed according to the seventh AJCC/UICC TNM classification system [36]. All of the surgeries were performed by surgeons who specialize in gastric cancer. The data were prospectively collected and were entered into a computer file, and the follow-up conditions of the patients were regularly updated.

Prior to surgery, all of the patients underwent chest radiography, abdominal sonography, or CT scanning for tumor staging. A total or distal subtotal gastrectomy was performed depending on the distance between the cardia and the tumor with a margin of 3 cm needed for superficial and well-defined tumors, and a margin of 5 cm needed for advanced or poorly defined tumors. A subtotal gastrectomy is the standard surgical procedure for distal gastric cancer, whereas a total gastrectomy is the more common surgical procedure for proximal gastric cancer.

### Follow-up

The overall survival was calculated from the time of surgery until death or the date of the last follow-up visit. None of the patients received preoperative chemotherapy. Before 2008, adjuvant chemotherapy or radiotherapy after curative surgery was not routinely given; rather, such therapies were given only when tumor recurrence was diagnosed or highly suspected. Since 2008, adjuvant therapy (such as S-1) has been prescribed for stage II or stage III disease in our hospital after curative surgery due to its proven survival benefit [37]. None of the 431 patients enrolled in this study received adjuvant therapy after surgery.

Follow-up assessments were performed every 3 months for the first 5 years after surgery and every 6 months thereafter until the patient's death. The follow-up procedures involved medical histories, physical examinations, routine blood tests, liver function tests, measurement of the levels of tumor markers (e.g., carcinoembryonic antigen and carbohydrate antigen 19-9), chest radiography, abdominal sonography, and CT scanning.

### Detection of mutations using a highly sensitive MALDI-TOF technology

Patients were considered to carry a PI3K/AKT pathway mutation when any one of the *PIK3CA*, *PTEN*, *AKT1*, *AKT2*, or *AKT3* genes contained a mutation. A total of 39 somatic mutations within the PI3K/AKT pathway genes were selected based on the frequency of their occurrence in tumor tissue according to the ‘Catalogue of Somatic Mutations in Cancer’ database [http://cancer.sanger.ac.uk/cancergenome/projects/cosmic/] (Supplementary Table 1). Mutation assays were performed using a Sequenom MassARRAY system (Sequenom, San Diego, CA, USA). The PCR and single-base extension primers were designed using MassARRAY Assay Design 3.1 software, and three multiplex reactions were designed to detect these 39 PI3K/AKT pathway-related mutations. PCR reactions were performed in a final volume of 5 μl containing 1 pmol of the appropriate primers, 10 ng of genomic DNA, and reaction mix (Sequenom) in 384-well plates. The conditions for the PCR reactions, which were performed using tumor DNA to amplify the regions that harbored loci of interest, were as follows: 94°C for 15 min, followed by 40 cycles at 94°C (20 s), 56°C (30 s), and 72°C (60 s), with a final extension at 72°C for 3 min. During the primer extension step, the PCR products were incubated with probes that immediately annealed adjacent to the mutation sites and single-base extension was conducted in the presence of chain-terminating di-deoxynucleotides that generated allele-specific DNA products. The single-base extension conditions were as follows: 94°C for 2 min, followed by 40 cycles at 94°C (5 s), 52°C (5 s), and 72°C (5 s). The extension products were spotted onto a SpectroCHIP II (Sequenom) and were then analyzed using matrix-assisted laser desorption ionization-time of flight (MALDI-TOF) mass spectrometry (Sequenom) to determine the mutational status based on the difference in the mass of the mutant and wild-type bases. Each spectrum was then analyzed using Typer 4.0 software (Sequenom) to perform variant genotype calling. Putative mutations were further filtered by manual review. To estimate the percentage of a mutant allele, the relative signal intensity was determined using the following equation: (mutant peak area)/(mutant peak area+ wild-type peak area). The sensitivity of the MALDI-TOF mutational assay varied because a specific assay was designed for each individual mutation. To generate different dilutions of each mutation, we obtained wild-type and mutant dsDNA (gBlocks gene fragments) from Integrated DNA Technologies (Coralville, IA, USA) and used the qPCR method to quantify the number of DNA copies in each gBlock DNA fragment for pooling. The sensitivity of the assay for each of the PI3K/AKT pathway mutations considered in this study was as low as 1-5% (Supplementary Table 3); cluster plots and representative spectra of selected dilutions are shown in Supplementary Figure 1.

### *PIK3CA* amplifications

The copy number of the *PIK3CA* gene was analyzed using qPCR, with the LINE1 element used as an internal reference target, using the primer employed in a previous study [38]. qPCR was then conducted on an LightCycler 480 II system (Roche Diagnostics, Mannheim, Germany) in a total volume of 20 μl of reaction mixture containing 10 μl of KAPA SYBR FAST qPCR Master Mix (Kapa Biosystems, Woburn, MA, USA), 8 pmol of each primer, and 10 ng of DNA. The PCR conditions were as follows: 95°C for 1 min, followed by 40 cycles of 95°C for 15 s, and 60°C for 30 s. The threshold cycle number (Ct) values of the *PIK3CA* gene and LINE1 were determined. The *PIK3CA* gene copy number was calculated using the delta-delta-Ct method in triplicate [39]. The relative copy number in each sample was determined by comparing the ratio of *PIK3CA* gene to the LINE1 element in each sample, and 20 normal human genomic DNA samples isolated from human blood cells were used to generate the diploid delta-Ct distribution. As previously reported [11], amplification of the *PIK3CA* gene was defined as a copy number of ≥ 3 with *P*-value of <0.05.

### Statistical analysis

The statistical analyses were performed using SPSS software (version 16.0 for Windows, SPSS, Chicago, IL, USA). All of the results in the text and tables are presented as the means values ± the standard deviations (SD). The categorical data were compared using a χ2 test with Yates correction or Fisher's exact test. The overall survival was measured from the date of the operation to the date of death or the final follow-up. The disease-free survival was defined as the length of time after gastric cancer surgery during which the patient survived without tumor recurrence. The distributions of overall survival and disease-free survival were estimated using the Kaplan-Meier method. Cox proportional hazards models were used to explore the association between the clinical parameters and the overall survival and disease-free survival. A *P* value of < 0.05 was considered statistically significant.

## SUPPLEMENTARY FIGURE AND TABLES


