# Supplementary material for: Mutations in PI3K/AKT pathway genes and amplifications of PIK3CA are associated with patterns of recurrence in gastric cancers
Source: Oncotarget. 2015 Dec 17;7(5):6201–20. doi: 10.18632/oncotarget.6641 (PMC4868750; doi:10.18632/oncotarget.6641)
Supplement: Supplementary file 1 [file oncotarget-07-6201-s001.pdf]

## SUPPLEMENTARY FIGURE AND TABLES

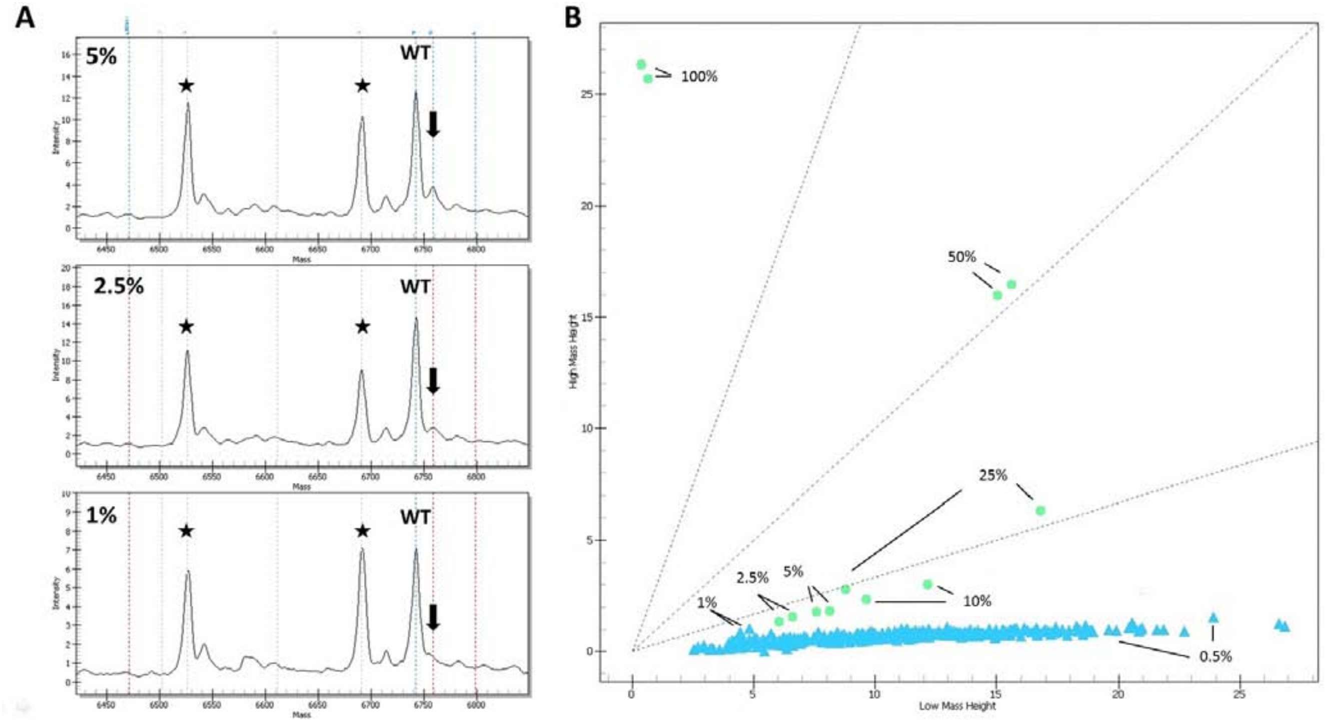


**Supplementary Figure S1: Sensitivity study for MALDI-TOF *PIK3CA* c.3140G>T mutation. A.** The raw MALDI-TOF spectrum of dilutions of 5%, 2.5%, and 1% mutant DNA was shown (x axis = mass, y axis = intensity). Arrow indicates *PIK3CA* c.3140G>A mutation mass location; WT indicates wild-type peak; asterisk indicates the peak from another assay in the multiplexed assay. **B.** The wild-type G (x axis) versus the mutant T (y axis) mass height cluster distribution at 0.5%, 1%, 2.5%, 5%, 10%, 25%, 50% and 100% dilutions in replicates (generated by pooling artificial synthesized WT and mutant dsDNA). Green dots indicate mutant calls, and blue dots include wild-type results.

**Supplementary Table S1: The 39 target mutations in 5 genes from the PI3K/AKT pathway**

| Gene | cDNA change | Amino acid mutation | Patient number |
|---|---|---|---|
| *AKT1* | c.49G>A | p.E17K | 1 |
| *AKT2* | c.1063C>T | p.H355Y | 1 |
| | c.904A>G | p.S302G | 1 |
| | c.1112G>A | p.R371H | 0 |
| *AKT3* | c.514A>C | p.K172Q | 0 |
| | c.496C>T | p.R166* | 2 |
| | c.49G>A | p.E17K | 4 |
| | c.1080G>A | p.M360I | 0 |
| | c.1100G>A | p.R367Q | 0 |
| | c.1192G>T | p.E398* | 0 |
| *PIK3CA* | c.263G>A | p.R88Q | 3 |
| | c.1035T>A | p.N345K | 1 |
| | c.1258T>C | p.C420R | 1 |
| | c.1616C>G | p.P539R | 0 |
| | c.1624G>A | p.E542K | 10 |
| | c.1633G>A | p.E545K | 14 |
| | c.2102A>C | p.H701P | 0 |
| | c.3140A>T | p.H1047L | 2 |
| | c.3140A>G | p.H1047R | 11 |
| | c.3139C>T | p.H1047Y | 1 |
| | c.1634A>C | p.E545A | 2 |
| | c.3204_3205insA | p.N1068fs*4 | 0 |
| | c.1634A>G | p.E545G | 2 |
| | c.3062A>G | p.Y1021C | 1 |
| | c.331A>G | p.K111E | 3 |
| *PTEN* | c.388C>T | p.R130* | 2 |
| | c.388C>G | p.R130G | 0 |
| | c.389G>A | p.R130Q | 1 |
| | c.517C>T | p.R173C | 2 |
| | c.518G>A | p.R173H | 3 |
| | c.697C>T | p.R233* | 1 |
| | c.1003C>T | p.R335* | 5 |
| | c.968_969insA | p.N323fs*2 | 0 |
| | c.968delA | p.N323fs*21 | 0 |
| | c.800delA | p.K267fs*9 | 5 |
| | c.951_954delACTT | p.V317fs*3 | 0 |
| | c.741_742insA | p.P248fs*5 | 0 |
| | c.389delG | p.R130fs*4 | 1 |
| | c.16_17delAA | p.K6fs*4 | 0 |

Some patients have more than one hotspot mutations

**Supplementary Table S2: The 11 patients with two hotspot genetic mutations in PI3K/AKT pathway**

| Patient No. | Age/ sex | Tumor location | TNM stage | EBV infection | PI3KCA | PTEN | AKT3 | Survival status |
|---|---|---|---|---|---|---|---|---|
| 1 | 74/M | Body/antrum | T3N1/IIB | - | - | c.800delA c.388C>T | - | Died of recurrence |
| 2 | 73/M | Antrum/body | T4aN0/IIB | - | - | c.517C>T c.518G>A | - | Died of recurrence |
| 3 | 47/F | Cardia | T3N3/IIIB | + | c.331A>G c.1624G>A | - | - | Died of recurrence |
| 4 | 69/M | Antrum | T3N0/IIA | - | c.3140A>G c.1633G>A | - | - | Alive without recurrence |
| 5 | 52/F | Antrum | T4bN0/IIIB | - | c.3140A>G | c.697C>T | - | Alive without recurrence |
| 6 | 70/M | Antrum/body | T4aN2/IIIB | - | c.1634A>G | c.389G>A | - | Died of recurrence |
| 7 | 78/M | Body | T4aN3/IIIC | + | c.1633G>A | c.800delA | - | Died of recurrence |
| 8 | 84/M | Body/antrum | T2N2/IIB | - | c.1624G>A | c.800delA | - | Alive without recurrence |
| 9 | 57/M | Antrum | T3N2/IIIA | - | - | c.517C>T | c.49G>A | Died of recurrence |
| 10 | 69/F | Antrum/body | T2N0/IB | - | - | c.800delA | c.49G>A | Died of hepatoma, no recurrence |
| 11 | 38/M | Body/antrum | T2N0/IB | - | c.3062A>G | - | c.496C>T | Recurrence s/p op, Alive |

**Supplementary Table S3: Sensitivity study for PI3K/AKT pathway mutations: Sequenom Assay**

| Gene | cDNA change | Detectable mutant calls in both the cluster plot and spectrum for serial dilutions of mutant DNA | | | | | | | |
|---|---|---|---|---|---|---|---|---|---|
| | | 100% | 50% | 25% | 10% | 5% | 2.5% | 1% | 0.5% |
| *AKT1* | c.49G>A | Yes | Yes | Yes | Yes | Yes | No | No | No |
| *AKT2* | c.1063C>T | Yes | Yes | Yes | Yes | Yes | No | No | No |
| | c.904A>G | Yes | Yes | Yes | Yes | Yes | No | No | No |
| | c.1112G>A | Yes | Yes | Yes | Yes | Yes | No | No | No |
| *AKT3* | c.514A>C | Yes | Yes | Yes | Yes | Yes | Yes | Yes | No |
| | c.496C>T | Yes | Yes | Yes | Yes | Yes | No | No | No |
| | c.49G>A | Yes | Yes | Yes | Yes | Yes | No | No | No |
| | c.1080G>A | Yes | Yes | Yes | Yes | Yes | No | No | No |
| | c.1100G>A | Yes | Yes | Yes | Yes | Yes | No | No | No |
| | c.1192G>T | Yes | Yes | Yes | Yes | Yes | No | No | No |
| *PIK3CA* | c.263G>A | Yes | Yes | Yes | Yes | Yes | No | No | No |
| | c.1035T>A | Yes | Yes | Yes | Yes | Yes | Yes | No | No |
| | c.1258T>C | Yes | Yes | Yes | Yes | Yes | No | No | No |
| | c.1616C>G | Yes | Yes | Yes | Yes | Yes | Yes | Yes | No |
| | c.1624G>A | Yes | Yes | Yes | Yes | Yes | No | No | No |
| | c.1633G>A | Yes | Yes | Yes | Yes | Yes | No | No | No |
| | c.2102A>C | Yes | Yes | Yes | Yes | Yes | Yes | Yes | No |
| | c.3140A>T | Yes | Yes | Yes | Yes | Yes | Yes | No | No |
| | c.3140A>G | Yes | Yes | Yes | Yes | Yes | No | No | No |
| | c.3139C>T | Yes | Yes | Yes | Yes | Yes | No | No | No |
| | c.1634A>C | Yes | Yes | Yes | Yes | Yes | No | No | No |
| | c.3204_3205insA | Yes | Yes | Yes | Yes | Yes | Yes | Yes | No |
| | c.1634A>G | Yes | Yes | Yes | Yes | Yes | No | No | No |
| | c.3062A>G | Yes | Yes | Yes | Yes | Yes | No | No | No |
| | c.331A>G | Yes | Yes | Yes | Yes | Yes | No | No | No |
| *PTEN* | c.388C>T | Yes | Yes | Yes | Yes | Yes | No | No | No |
| | c.388C>G | Yes | Yes | Yes | Yes | Yes | No | No | No |
| | c.389G>A | Yes | Yes | Yes | Yes | Yes | No | No | No |
| | c.517C>T | Yes | Yes | Yes | Yes | Yes | No | No | No |
| | c.518G>A | Yes | Yes | Yes | Yes | Yes | Yes | Yes | No |
| | c.697C>T | Yes | Yes | Yes | Yes | Yes | No | No | No |
| | c.1003C>T | Yes | Yes | Yes | Yes | Yes | No | No | No |
| | c.968_969insA | Yes | Yes | Yes | Yes | Yes | No | No | No |
| | c.968delA | Yes | Yes | Yes | Yes | Yes | No | No | No |
| | c.800delA | Yes | Yes | Yes | Yes | Yes | No | No | No |
| | c.951_954delACTT | Yes | Yes | Yes | Yes | Yes | Yes | Yes | No |
| | c.741_742insA | Yes | Yes | Yes | Yes | Yes | No | No | No |
| | c.389delG | Yes | Yes | Yes | Yes | Yes | No | No | No |
| | c.16_17delAA | Yes | Yes | Yes | Yes | Yes | Yes | Yes | No |

Yes, Mutant calls detected in both the cluster plot and spectrum;
No, Mutant call not detected in either one or both of the cluster plot and spectrum.
